# Comparative transcriptomic analysis of *streptococcus pseudopneumoniae* with viridans group streptococci

**DOI:** 10.1186/1471-2180-12-77

**Published:** 2012-07-06

**Authors:** Hee Kuk Park, Soon Chul Myung, Wonyong Kim

**Affiliations:** 1 Department of Microbiology & Research Center for Medical Sciences, Chung-Ang University College of Medicine, Seoul, 156-756, Republic of Korea; 2 Department of Urology, Chung-Ang University College of Medicine, Seoul, 156-756, Republic of Korea

## Abstract

**Background:**

*Streptococcus pseudopneumoniae*, is a novel member of the genus *Streptococcus*, falling close to related members like *S. pneumoniae, S. mitis*, and *S. oralis*. Its recent appearance has shed light on streptococcal infections, which has been unclear till recently. In this study, the transcriptome of *S. pseudopneumoniae* CCUG 49455^T^ was analyzed using the *S. pneumoniae* R6 microarray platform and compared with those of *S. pneumoniae* KCTC 5080^T^, *S. mitis* KCTC 3556^T^, and *S. oralis* KCTC 13048^T^ strains.

**Results:**

Comparative transcriptome analysis revealed the extent of genetic relatedness among the species, and implies that *S. pseudopneumoniae* is the most closely related to *S. pneumoniae.* A total of 489, 444 and 470 genes were upregulated while 347, 484 and 443 were downregulated relative to *S. pneumoniae* in *S. pseudopneumoniae*, *S. oralis* and *S. mitis* respectively. Important findings were the up-regulation of TCS (two component systems) and transposase which were found to be specific to *S. pseudopneumoniae*.

**Conclusions:**

This study provides insight to the current understanding of the genomic content of *S. pseudopneumoniae.* The comparative transcriptome analysis showed hierarchical clustering of expression data of S*. pseudopneumoniae* with *S. pneumoniae* and *S. mitis* with *S. oralis.* This proves that transcriptional profiling can facilitate in elucidating the genetic distance between closely related strains.

## Background

*Streptococcus pseudopneumoniae* is a recently described member of the ‘*S. mitis*’ group of viridians streptococci, which is phenotypically and genetically close to *S. pneumoniaeS. mitis*, and *S. oralis*[1]. *S. pseudopneumoniae* strains characterized to date has been isolated from the lower respiratory tract [2-4]. This species is known to cause infections in patients having a history of chronic obstructive pulmonary disease or exacerbation of chronic obstructive pulmonary disease [4,5]. However, the clinical significance of this species is currently unknown.

*Streptococcus pneumoniae* is the most common cause of well-defined clinical syndrome of pneumonia, bacterial meningitis, and nongonoccal urethritis in humans [6-8]. By contrast, two medically important ‘*S. mitis*’ group streptococci, *S. mitis* and *S. oralis* are recognized as important etiological agents for subacute endocarditis and septicaemia [9,10]. Recently, pancreatic cancer has been associated with *S. mitis*, increasing the clinical relevance of this group [11].

The pathogenicity and the underlying genetic identity of *S. pseudopneumoniae* are not well characterized in relation to its phylogenetic neighbours, *S. pneumoniae, S. mitis,* and *S. oralis.* Unlike *S. pneumoniaeS. pseudopneumoniae* is optochin resistant in the presence of 5% CO_2_, is bile insoluble, and lacks the pneumococcal capsule [12,13]. The use of MLST described in this paper allowed a good differentiation between the species [14]. In clinical studies, the phenotypic characterization of the isolates showed relatedness to the species *S. pseudopneumoniae*, but genotypically it was difficult to distinguish from its close neighbour *S. pneumoniae*[1]. Indeed, *S. pseudopneumoniae* shares over 99% 16S rRNA gene homology with *S. pneumoniae, S. mitis,* and *S. oralis*[15] showing that it has evolved from a common genetic ancestor [16-18]. In recent years, several reports have shown that *S. pneumoniae* share genes encoding virulence factors with *S. mitis* and *S. oralis*, providing suggestive evidence of lateral gene transfer between these species [19,20].

Genotypic characterization of *S*. *pseudopneumoniae* in relation to its neighboring members is necessary to increase its clinical relevance. Comparative genomics or transcriptomics based on genome wide microarrays [21], is now the logical approach used to determine inter-species comparisons [22,23]. Since whole-genome sequencing to elucidate the genetic content of a microorganism is considered to be expensive and time consuming, an approach used for the identification of large number of genes without the need for sequencing is the trend in present era. The entire genomes of *S. pneumoniaeS. mitis*, and *S. oralis* have been fully sequenced. However, transcriptome has not been studied in these microorganisms to date, which may lead to the identification of unique virulence genes specific to the strain of interest.

Previously, we identified species-specific genes using suppressive subtractive hybridization (SSH), such as the *cpsA* gene for *S. pneumoniae* and the *rgg* gene for *S. oralis*[24-26]. In the current study, the gene expression of *S. pseudopneumoniae* is determined and compared with those of *S. pneumoniae* KCTC 5080^T^*S. mitis* KCTC 3556^T^ and *S. oralis* KCTC 13048^T^ by *in silico* analysis and by *in vitro* transcriptome microarrays experiments using open reading frame (ORF) microarrays of *Streptococcus pneumoniae* R6 (GenBank accession number NC_003098) platform.

## Results and discussion

### Statistical analysis of microarray experiments

**Figure 1:**
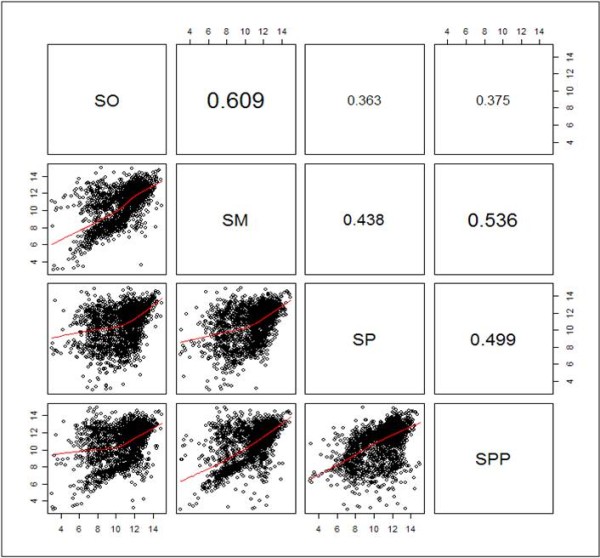
**Reproducibility and dynamic range with pairwise scatter plots.** Four technical replicates of *Streptococcus pseudopneumoniae*, *Streptococcus pneumoniae, Streptococcus mitis*, and *Streptococcus oralis* RNA were hybridized to NimbleGen *Streptococcus pneumoniae* R6 Gene Expression 4x72K microarrays. Interarray correlation values (Range: -1 ≤ r ≤ 1) are shown in the upper right panels and pairwise scatter plots of gene expression values (log2) are shown in the lower left panels. So, *S. oralis*; Sm, *S. mitis*; Spp, *S. pseudopneumoniae*; Sp: *S. pneumoniae*

We compared the expression profiles by hybridization to the immobilized probes on the microarray of *S. pneumoniae* TIGR4: NC_003028 with the total RNA of *S. oralis* KCTC 13048^T^, *S. mitis* KCTC 3556^T^, and *S. pseudopneumoniae* CCUG 49455^T^. Total RNA from the strains *S. pneumoniae* KCTC 5080^T^, *S. mitis* KCTC 3556^T^, *S. oralis* KCTC 13048^T^, and *S. pseudopneumoniae* CCUG 49455^T^ was hybridized to NimbleGen *S. pneumoniae* TIGR4: NC_003028 Gene Expression 4x72K microarrays. Each array contains 4 sets of strains, and each strain was compared with each other strains. Interarray correlation values (Range: -1 ≤ r ≤ 1) are shown in the upper right panels and pairwise scatter plots of gene expression values (log2) are shown in the lower left panels (Figure 1). A correlation value close to 1 shows high similarity between samples. This correlation value between strains *S. oralis-S. mitis* was 0.609, *S. oralis-S. pneumoniae* was 0.365, S*. oralis-S. pseudopneumoniae* was 0.375, *S. mitis-S. pneumoniae* was 0.438, *S. mitis-S. pseudopneumoniae* was 0.536 and *S. pneumoniae-S. pseudopneumoniae* was 0.499.

### Phylogenetic relatedness between streptococcal species

**Figure 2:**
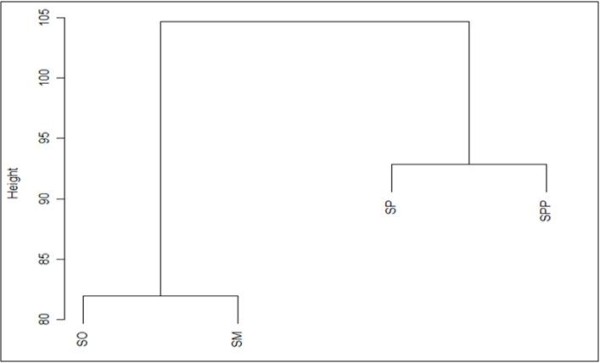
**Hierarchical clustering.** Phylogenetic relationships of *S. mitis, S. oralis*, *S. pneumoniae*, and *S. pseudopneumoniae*. Each strain formed distinct branch and was placed in a separate two clusters. *S. pseudopneumoniae* is more closely related to *S. pneumoniae*. So, *S. oralis*; Sm, *S. mitis*; Spp, *S. pseudopneumoniae*; Sp: *S. pneumoniae*

**Figure 3:**
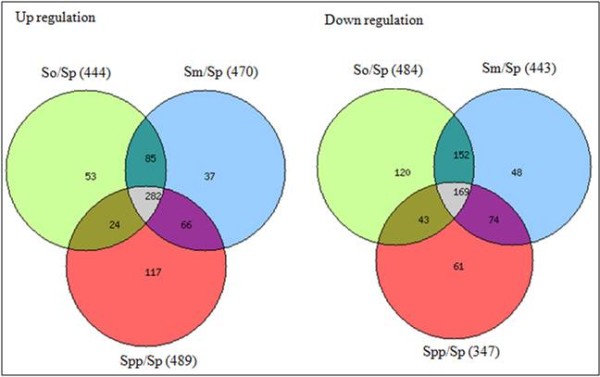
**Venn diagram of gene in the viridians group streptococci.** So, *S. oralis*; Sm, *S. mitis*; Spp, *S. pseudopneumoniae*; Sp: *S. pneumoniae*

**Figure 4:**
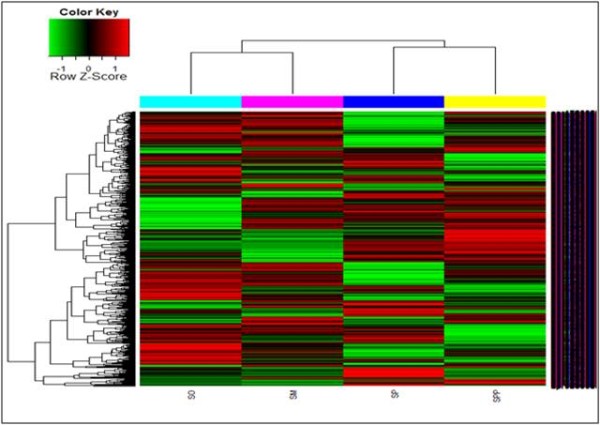
**The map of hierarchical clustering of differentially expressed genes.** Red represents high expression; green represents low expression. So, *S. oralis*; Sm, *S. mitis*; Spp, *S. pseudopneumoniae*; Sp: *S. pneumoniae*

Based on their overall genomic profiles, there was clear delineation between each *Streptococcus* species. The hierarchical clustering analysis from a normalized signal grouped the isolates mainly according to their phylogenetic relationship between each *Streptococcus* species. The clustering of *S. mitis, S. oralis* and *S. pneumoniae*, *S. pseudopneumoniae* strains showed two distinct branches, placing them in two separate clades that clearly differentiated each species group (Figure 2). The map shows the expression levels of the 1,123 probes (Figure 3). A total of 444 genes were upregulated (red) and 484 genes were downregulated(green) in *S. oralis* KCTC 13048^T^, 470 genes were upregulated (red) and 443 genes were downregulated (green) in *S. mitis* KCTC 3556^T^ and 489 genes were upregulated (red) and 347 genes were downregulated (green) in *S. pseudopneumoniae* CCUG 49455^T^ (Figure 3). Red represents high expression; green represents low expression (Figure 4).

### Identification of functional genes revealed by transcriptome analysis

Whole-genome sequence of *S. pseudopneumoniae* (isolate number: IS7493, GenBank accession numbers: CP002925 and CP002926) was done by Shahinas *et al.*[27]. Their study shows the presence or absence of genes in the whole genome but not the functional analysis of RNA transcripts. In this study, the availability of the complete *S. pneumoniae* TIGR4: NC_003028 genome [28] allowed for the analysis of *S. oralis* KCTC 13048^T^ total RNA transscripts.

About 53 genes were up regulated in *S. oralis* KCTC 13048^T^ when compared with other *Streptococcus* species (Table 1). About 26 genes were identified as hypothetical proteins while the remaining 27 were associated with amino acid biosynthesis, transport and degenerate transposase proteins.

**Table 1:** **Up regulated genes from the*S. oralis, S. mitis*, and*S. pseudopneumoniae***

**Gene name**	**Function**	***S. oralis***	***S. mitis***	***S. pseudopneumoniae***
spr1541	4'-phosphopantetheinyl transferase	-	1	-
spr0535	ABC transporter ATP-binding protein	6	3	6
spr0853	Alanine dehydrogenase	2	-	-
spr0262	alcohol dehydrogenase	-	1	-
spr0247	Alkaline amylopullulanase	-	1	-
spr0307	ATP-dependent protease	1	-	-
spr1862	Competence protein	1	-	-
spr0469	Conserved hypothetical protein	-	-	4
spr0369	D-alanine glycine permease	-	-	1
spr1563	Degenerate transposase	1	2	11
spr0227	DEOR-type transcriptional regulator	1	-	-
spr0347	DNA alkylation repair enzyme, truncation	1	-	-
spr1144	DNA processing Smf protein	-	1	-
spr1088	Exodeoxyribonuclease VII small subunit	1	-	-
spr0136	Glycosyl transferase, family 2	1	-	-
spr1894	Histidine kinase	-	-	1
spr1326	Hypothetical protein	26	23	54
spr1453	Major facilitator superfamily transporter - efflux?	-	-	1
spr1922	Maltose operon transcriptional repressor	-	-	1
spr0356	Mannitol PTS EII	-	-	1
spr0359	Mannitol-1-phosphate 5-dehydrogenase	-	-	1
spr0358	Mannitol-specific enzyme IIA component	-	-	1
spr0647	Mannose-6-phosphate isomerase	1	-	-
spr0696	Methionine--tRNA ligase	1	-	-
spr1714	MSM (multiple sugar metabolism) operon regulatory protein	-	1	-
spr1323	NADH oxidase	1	-	-
spr1899	Negative regulator of pho regulon for phosphate transport	-	-	1
spr1095	O-acetylhomoserine sulfhydrylase, truncation	1	-	-
spr0127	orf51	-	-	1
spr1333	Peptidoglycan GlcNAc deacetylase	-	-	1
spr0241	Phosphatidate cytidylyltransferase	-	1	-
spr0562	Phosphotransferase system sugar-specific EII component	2	-	1
spr0140	Positive transcriptional regulator of mutA	-	-	1
spr1718	RecA regulator RecX	-	1	-
spr1107	Response regulator	-	-	1
spr0164	Riboflavin biosynthese; a deaminase	-	-	2
spr0163	riboflavin synthase subunit alpha	-	-	1
spr1098	Sortase	-	-	1
spr1771	Subtilisin-like serine protease	1	-	-
spr1878	threonine synthase	1	-	-
spr1051	TPP-dependent acetoin dehydrogenase alpha chain	2	-	-
spr0842	Transposase	-	-	18
spr1803	Transcriptional activator	-	-	1
spr0504	Transcriptional antiterminator	-	-	1
spr0044	Transport protein ComB	-	1	-
spr1700	Trehalose operon transcriptional repressor	-	-	1
spr0362	trigger factor	1	-	-
spr0071	Trk transporter NAD + binding protein - K + transport	-	-	1
spr0687	tRNA (guanine-N(1)-)-methyltransferase	1	-	-
spr1900	truncated IS1380-Spn1 transposase	-	-	1
spr0792	Type 1 restriction modification system endonuclease R	-	-	1
spr0790	Type I restriction modification enzyme methylase subunit	-	-	1
spr1683	UDP-galactose 4-epimerase, truncation	-	1	-

The 37 genes differentially regulated in *S. mitis* KCTC 3556^T^ were found to function in amino acid biosynthesis, transport and were transposases, including 4’-phosphopantetheinyl transferase, ABC transporter, alcohol dehydrogenase, alkaline amylopullulanase, Smf DNA processing protein, MSM (multiple sugar metabolism) operon regulatory protein, Peptidoglycan GlcNAc deacetylase, Phosphatidate cytidylyltransferase, *RecA* regulator *RecX*, Transport protein *ComB*, UDP-galactose 4-epimerase, truncation, as well as other hypothetical proteins (Table 1).

The 117 upregulated genes of *S. pseudopneumoniae* CCUG 49455^T^, were found to play a role in amino acid biosynthesis and transport, such as ABC transporter ATP-binding protein, conserved hypothetical protein, D-alanine glycine permease, histidine kinase, major facilitator superfamily transporter, maltose operon transcriptional repressor, mannitol PTS EII, mannitol-1-phosphate 5-dehydrogenase, mannitol-specific enzyme IIA component, negative regulator of pho regulon for phosphate transport, peptidoglycan GlcNAc deacetylase, phosphotransferase system, positive transcriptional regulator of mutA, response regulator, riboflavin synthase, sortase and transcriptional proteins.

The degenerate transposon was significantly overexpressed in *S. pseudopneumoniae* compared to its expression in *S. oralis* and *S. mitis*. On the other hand, histidine kinase and response regulators associated with the two component system (TCS) were down regulated in the *S. oralis* and *S. mitis* (Table 1). Additionally pneumolysin and penicillin-binding protein were also down regulated in *S. oralis* and *S. mitis* and showed no signal in the *S. pseudopneumoniae.*

Upregulation of some interesting genes in the transport group was found in *S. pseudopneumoniae* like the ATP-binding cassette (ABC) transporters and the two component system (TCS). ABC transporters are integral membrane proteins that actively transport chemically diverse substrates across the lipid bilayers of cellular membranes. This is of clinical importance because multidrug resistance in human cancer cells is mostly the result of the over expression of ABC transporters that catalyze the extrusion of the cytotoxic compounds used in cancer therapy [29]. Bacterial drug resistance has become an increasing problem. In bacterial cells, ABC transporters are known to contribute to multidrug and antibiotic resistance by extruding drugs or antibiotics [30].

The TCSs of bacteria consist of two proteins, histidine kinase and response regulators, and have received increasing attention for their potential as a novel antibacterial drug targets [31,32]. Some TCSs regulate the expression of antibiotic resistance determinants, including drug-efflux pumps [33]. The overexpression of response regulators of bacterial two-component signal transduction system confers drug resistance by controlling the expression of some drug transporter genes. Various TCSs ubiquitously present in bacteria regulate the transcription of different gene products. The regulation of osmolarity, nutrient uptake, redox potential, sporulation and the expression of virulence factors are under the control of TCSs. The two component system (TCS) serves as a basic stimulus–response coupling mechanism that allows organisms to sense and respond to changes in environmental conditions. The sensor kinase monitors a certain environmental condition and modulates the phosphorylation state of the response regulator that controls genes. One of the most attractive aspects of the TCS is its regulation of antimicrobial resistance factors.

## Conclusions

In summary, based on comparative genomics/transcriptome analysis, using *S. pneumoniae* as the control strain, facilitated the identification of *S. pseudopneumoniae* transcriptome within streptococci viridans group. We postulate that transcriptional profiling with high statistical power implies the great genetic distance between each streptococci of viridans group. The correlation values by statistical analysis show the closest association between *S. oralis* and *S.mitis*. This is also clearly shown by the clustering method which placed *S.oralis* and *S.mitis* in a separate clade from *S.pneumoniae* and *S. pseudopneumoniae* revealing their genetic relatedness. Overall expression levels of 489 genes were higher in *S.mitis* strain when compared with the control strain. Some of the important genes identified by functional analysis at RNA level were those belonging to amino acid biosynthesis, transport and degenerate transposase proteins. One of the significant findings in this study was the upregulation of ABC transporters and TCS in *S. pseudopneumoniae* where the former are known to play a role multi-drug antibiotic resistance and the latter in controlling the virulence factors. Therefore, we conclude by this study that genetic relatedness and pathogenecity in *S. pseudopneumoniae* in comparison to viridans group was well revealed by transcriptome analysis.

## Methods

### Bacterial culture, RNA extraction and cDNA synthesis

*S. pneumoniae* KCTC 5080^T^ was used as the reference strain for comparative microarray experiments with other viridians group of streptococci. *S. pneumoniae* KCTC 5080^T^, *S. pseudopneumoniae* CCUG 49455^T^, *S. mitis* KCTC 3556^T^, and *S. oralis* KCTC 13048^T^ strains were grown on Brain Heart Infusion (BHI) agar (Difco, Detroit, MI, U.S.A.) at 37°C for 18 hours. Total RNA was isolated using a RiboPure Bacteria Kit (Ambion, UK) following manufacturer’s instructions. Extracted RNA was treated with TURBO DNase (Ambion). RNA quality was checked for purity and integrity as evaluated by OD 260/280 ratio, and analyzed on Agilent 2100 Bioanalyzer (Agilent Technologies, Palo Alto, USA). cDNA was synthesized according to the NimbleGen Expression protocol (Nimblegen, Madison, USA) using the SuperScript double-stranded cDNA synthesis kit (Invitrogen Life Technologies, Carlsbad, CA, U.S.A.). Briefly, 10 μg of total RNA was reverse-transcribed to cDNA using an oligo dT primer. Then second-strand cDNA was synthesized. After purification, cDNA was quantified using the ND-1000 Spectrophotometer (NanoDrop, Wilmington, USA).

### Labeling and purification

cDNA was labelled using the One-Color Labelling Kit (Nimblegen) following manufacturer’s instructions. 1 μg of cDNA samples were labelled with Cy3 using Cy3-random nonamer. After purification, the labelled cDNA was quantified using the ND-1000 Spectrophotometer (NanoDrop).

### Generation of microarray data

The *Streptococcus pneumoniae* R6 microarrays (Nimblegen) were used for the transcriptome analysis. The *S. pneumoniae* R6 microarray contains 2,037 genes: 4 × 72,000 probes and 5 replicates (GenBank accession numbers: NC_003098). Labelled cDNA samples of *S. pseudopneumoniaeS. mitis* and *S. oralis* were hybridized onto Nimblegen Expression array (Nimblegen) for 16-20 hours at 42°C, according to manufacturer's instructions. Arrays were scanned with a NimbleGen MS 200 Microarray scanner set- at 532 nm with a resolution of 2 μm to produce images in TIFF format according to the manufacturer's instructions. Array data export processing and analysis was performed using NimbleScan (version 2.5). The data discussed in this publication have been deposited in NCBI’s Gene Expression Omnibus [34] and are accessible through GEO Series accession number GSE37539 (http://www.ncbi.nlm.nih.gov/geo/query/acc.cgi?acc=GSE37539).

### Data acquisition and statistical analysis

Raw data was extracted using NimbleScan (version 2.5, Gene Expression RMA algorithm). A single raw intensity value was determined for each gene in each array with 2535 genes by taking an average of spot replicates of all 24 probes. Gene signal value was determined by logarithmic transformation (base 2). Statistical significance of the expression data was determined using fold change. Hierarchical cluster analysis was performed using complete linkage and Euclidean distance as a measure of similarity. NimbleScan was used for quantification, image analysis of mRNA data. R scripts (‘R’ software) were used for all other analytical process.

## Authors’ contributions

WK and SCM contributed to the design of experiments. HKP implemented experiments and drafted the manuscript. WK analyzed results and edited the manuscript. All authors read and approved the final manuscript.
